# Use of Optical Coherence Tomography to Detect Retinal Nerve Fiber Loss in Children With Optic Pathway Glioma

**DOI:** 10.3389/fneur.2018.01102

**Published:** 2018-12-20

**Authors:** Alon Zahavi, Helen Toledano, Rony Cohen, Sara Sella, Judith Luckman, Shalom Michowiz, Nitza Goldenberg-Cohen

**Affiliations:** ^1^Department of Ophthalmology, Rabin Medical Center-Beilinson Hospital, Petah Tikva, Israel; ^2^Sackler Faculty of Medicine, Tel Aviv University, Tel Aviv, Israel; ^3^Department of Pediatric Oncology, Schneider Children's Medical Center of Israel, Petah Tikva, Israel; ^4^Department of Pediatric Neurology and Epilepsy Center, Schneider Children's Medical Center of Israel, Petah Tikva, Israel; ^5^Radiology, Rabin Medical Center-Beilinson Hospital, Petah Tikva, Israel; ^6^Department of Neurosurgery, Schneider Children's Medical Center of Israel, Petah Tikva, Israel; ^7^Krieger Eye Research Laboratory, Felsenstein Medical Research Center, Petah Tikva, Israel; ^8^Department of Ophthalmology, Bnai Zion Medical Center, Haifa, Israel; ^9^Rappaport Faculty of Medicine, Technion, Israel Institute of Technology, Haifa, Israel

**Keywords:** optic pathway glioma, children, optical coherence tomography, OCT, neurofibromatosis 1, non-NF1

## Abstract

Optic pathway glioma (OPG) presents in childhood and can cause significant morbidity and visual loss. Magnetic resonance imaging (MRI) is the current imaging modality of choice for evaluation of OPG progression, but it is a relatively limited resource often requiring sedation in the pediatric age group. Additionally, OPG progression on MRI does not always correlate with clinical progression. As a result, several other modalities for evaluating OPG are being investigated, including optical coherence tomography (OCT), a readily available imaging technique in ophthalmic practice. The purpose of the present study was to examine the association between retinal nerve fiber layer (RNFL) thickness measured using OCT and optic nerve function in children with OPG with and without neurofibromatosis-1 (NF-1). A retrospective chart review was conducted to identify children diagnosed with OPG from 2001 to 2015 at a tertiary pediatric medical center. The correlation between OCT measurements and clinical visual parameters was statistically analyzed. Included were 23 children with imaging-confirmed OPG and spectral domain OCT: 10 with NF-1 (mean age at diagnosis 5.8 years) and 13 without (mean age at diagnosis 5.9 years). The glioma involved the chiasma-hypothalamus in 19 patients, optic nerve in 11, and optic tract in 7; more than one anatomic site was affected in 15. Symptoms were reported in 2 patients with NF-1 and most patients without NF-1. Visual field defects included monocular, bitemporal, nasal, and homonymous hemianopia. Initial mean RNFL was 85.4 μm in the NF-1 group and 65 μm in the non-NF-1 group. Visual acuity deteriorated in 1/10 patients and 5/13 patients, respectively. Repeated OCT showed continued RNFL thinning in 3 patients (5 eyes) in the NF-1 group and in 8 patients (11 eyes) in the non-NF-1 group, often associated with a decrease in optic nerve function. In conclusion, visual function in children with OPG is correlated with repeated OCT measurements and weakly with neuroimaging. Children without NF-1 are usually symptomatic and have a worse clinical outcome. These findings may have important implications when considering initiating, continuing or stopping chemotherapy for OPG. The application of OCT in the assessment of OPG and the correlation of the findings to clinical progression can have a significant impact on OPG patient management.

## Introduction

Optic pathway gliomas (OPGs) are histologically low-grade pilocytic astrocytomas which tend to appear in the first year of life. Many show prolonged indolent phases; spontaneous tumor regression has been documented as well (1–3). In rare cases, malignant astrocytomas may involve the anterior visual system (4). Although OPGs are usually associated with a high survival rate (5), they pose a high risk of significant morbidity and visual loss over time.

At diagnosis of OPG, visual function ranges from good to poor (6, 7). The clinical presentation usually correlates with the anatomic location (8) and size of the tumor (8, 9). Because monocular visual loss is not often diagnosed immediately, especially in children, the findings leading to diagnosis are usually proptosis or lack of stereopsis as well as failure on routine vision screening (9). Strabismus may also be a presenting sign (10). In patients with reduced vision, fundus examination may identify optic pallor (11).

The presence of neurofibromatosis-1 (NF-1) is a risk factor for the development of OPG (12, 13). Children with NF-1 are predisposed to the development of OPGs (14) at reported rates of 8–31% (5, 15, 16), and they are usually screened clinically (not radiologically) for OPGs on an annual basis. This is important because unlike children without NF-1, children with NF-1 are usually asymptomatic. The course of sporadic OPGs is more severe than NF-1-associated OPGs (14, 17), with more significant deterioration of vision (15).

Previous studies of treatment outcomes of OPG have focused mainly on changes in tumor size or patient survival, with treatment limited to patients exhibiting tumor growth on imaging or severe clinical symptoms, as estimated by clinical examination or visual evoked potentials (17). However, treatment to reduce tumor size does not necessarily affect visual function, and only recently has preservation of vision become a primary treatment objective (18–21). This has created a need to reliably follow changes in visual function in the pediatric population.

Optical coherence tomography (OCT) is a noninvasive interferometry-based ocular imaging technique. By measuring the backscatter of infrared light, the device generates high-resolution (within 4microns) cross-sectional images of the retinal nerve fiber layer (RNFL) (22). It is a useful tool for following thinning of the RNFL around the disc. Data on the value of OCT measurements in children with OPG, with or without NF-1, are limited. Several studies have investigated OCT-based parameters such as RNFL thickness and ganglion cell layer-inner plexiform layer thickness in children with OPG in terms of the correlation of longitudinal changes over time and visual acuity (23–31). Others compared RNFL changes with OPG tumor size (32). However, none of the previous studies compared sporadic and NF1-associated OPGs, combined with the radiological findings and the clinical and OCT-based findings.

The aim of the present study was to evaluate RNFL thickness in children with and without NF-1 who were diagnosed with OPG and to analyze the association of anatomical structural changes in tumor size and RNFL thickness with clinical changes in optic nerve function.

## Methods

A retrospective comparative cohort study design was used. The clinical database of the neurology and ophthalmology clinics of a tertiary university-affiliated pediatric medical center was reviewed for all children (age up to 18 years) with or without NF-1 who were diagnosed with OPG from 2001 through 2015 and underwent at least one spectral domain OCT (SD-OCT) scan of the optic nerve. The diagnosis of OPG in our departments is based on clinical as well as neuroimaging findings.

Data on demographic parameters, age at initial presentation, presenting symptoms, and signs, and visual function were derived from the medical files. Parameters of visual function were visual acuity, color vision (with Ishihara color plates), relative afferent pupillary defect (RAPD), and optic disc appearance. Visual acuity was measured for each eye separately and converted to logMAR units and categorized as normal (logMAR ≤ 0.3, equivalent to 20/40), moderate (logMAR > 0.3 and < 1.0), poor (logMAR ≥ 1.0, equivalent to 20/200), or no light perception (NLP). In the youngest patients, visual acuity was categorized as presence or absence of fix- and-follow. Optic disc appearance was categorized as normal, optic disc pallor (unhealthy pale appearance of the optic disc), or disc atrophy (clinically noticeable tissue loss at the optic disc). Findings for visual field test (confrontation for children under age 5 years, computerized for older children) were categorized as left/right monocular, temporal, or homonymous loss. In addition, the neuroimaging scans of all eligible patients were reviewed to identify the presence, location, and size of the tumor at diagnosis and follow-up, and the data were recorded. OCT was introduced in our center in 2007 for use in children who were able to cooperate. Some of the initial scans were performed with time domain (TD) technology (Stratus OCT, version 4.0.1, Carl Zeiss Meditec, Dublin, CA, USA) which was the standard in clinical practice at the time. Later scans were performed with a spectral domain (SD) OCT (Cirrus 4000, Carl Zeiss Meditec) which provides higher quality scans and has become the standard in recent years. In the present study, only the SD-OCT scans were included in the final analysis; all were performed with the same SD-OCT machine and by the same operator. The findings were analyzed for the whole cohort, for patients with and without NF-1, and over time.

### Statistical Analysis

Differences between groups in visual fields abnormalities, OCT results, and optic disc appearance were analyzed by Fisher's Exact Test, due to the small sample size. Spearman correlation was used to analyze the relationship between RNFL thickness and visual acuity, color vision, visual fields, or optic disc appearance. Non-parametric Wilcoxon was used to evaluate the effect of OPG location on RNFL thickness. Statistical significance was defined as *P* < 0.05.

## Results

Of the 59 patients with OPGs who attended the neurology and ophthalmology clinics of our medical center during the study period, 23 met the study criteria:10 with NF-1 (6 boys, 4 girls) and 13 without NF-1 (8 boys, 5 girls). Their characteristics are shown in Table 1. Mean age at diagnosis of OPG was 5.8 years in the NF-1 group (range 3–10 years) and 5.9 years (range 0.8–18) in the non-NF-1 group. In the NF-1 group, OPG was detected mostly by routine imaging conducted during routine follow up. One patient presented with proptosis and another with optic nerve pallor noted on routine screening. In the non-NF-1 group, the diagnosis was suspected on the basis of symptoms and signs in most patients. These included visual disturbances, headaches, hypothalamic obesity, proptosis, nystagmus, and strabismus. In the NF-1 group, the tumor involved the hypothalamus in 4 patients (40%), optic chiasma in 7 (70%), optic tract in 4 (40%), and optic nerve in 8 (80%). In the non-NF-1 group, the tumor involved the hypothalamus in 8 patients (61%), optic chiasma in 8 (61%), optic tract in 3 (23%), and optic nerve in 3 (23%). More than one anatomic location was involved in 7 patients with NF-1 and 8 patients without NF-1. The mean duration of follow-up was 81.6 months in the NF-1 group and 66.5 months in the non-NF-1 group.

**Table 1:** Clinical characteristics of patients with and without NF-1.

**Characteristics**	**NF-1**	**Non-NF-1**
**PATIENTS**		
Total	10	13
Boys	6	8
Girls	4	5
Age at diagnosis (year), range	3–10	0.8–18
Follow-up months, range (mean)	36–120 (81.6)	12–96 (66.5)
**PRESENTING SIGNS AND SYMPTOMS**		
Headache	0	4
Vomiting	0	4
Visual symptoms	0	7
Endocrine abnormalities	0	1
Proptosis	1	2
Nystagmus	0	2
Strabismus	0	3
Café au lait spots	8	0
Optic nerve pallor	1	0
**INITIAL VISUAL FIELD (LAST VISUAL FIELD)**		
Normal	7 (5)	2 (0)
Monocular	1 (2)	1 (0)
Bitemporal	1 (2)	2 (2)
Nasal	0 (0)	1 (1)
Homonymous	0 (0)	4 (6)
**INITIAL VISUAL ACUITY (LAST VISUAL ACUITY)**^†^		
F+F	1 (0)	1 (0)
Normal	7 (8)	4 (4)
Moderate	0 (0)	2 (1)
Poor	1 (1)	4 (4)
NLP	1 (1)	3 (4)
**LOCATION OF GLIOMA**		
Chiasma-hypothalamus	7	12
Optic nerve	8	3
Optic tract	4	3
Combined	7	8

† *Visual acuity was measured for each eye separately and converted to logMAR units and categorized as normal (logMAR ≤ 0.3, equivalent to 20/40), moderate (logMAR > 0.3 and < 1.0), poor (logMAR ≥ 1.0, equivalent to 20/200), or no light perception (NLP). In the youngest patients, visual acuity was categorized as presence or absence of fix and follow (F+F)*.

Treatment in all cases was based on the clinical course. Twelve children without NF-1 underwent chemotherapy, often combined with surgical intervention. The one exception was a patient diagnosed at age 18 years who had a stable clinical course following placement of an intraventricular shunt. Only 3 patients in the NF-1 group received chemotherapy during follow-up; none required surgical intervention.

### Visual Function

On initial visual field examination of the NF-1 group, 7 patients had normal visual fields, 1 had monocular defects and 1 had a bitemporal defect. One child had NLP at presentation, and therefore no visual field result. In the non-NF-1 group, of the 10 patients for whom data were available, 2 had normal visual fields, 1 monocular defects, 2 temporal defects, 1 nasal defects, and 4 homonymous defects. Three non-NF-1 children had NLP at presentation. At the end of the follow-up, visual fields were normal in 5 eyes in the NF-1 group; the remainder had monocular or temporal defects (2 patients each). One patient had NLP. In the non-NF-1 group, bitemporal defects were found in 2 patients, nasal defects in 1 patient, and homonymous defects in 6 patients. Four patients had NLP.

Findings on initial visual acuity testing were as follows: NF-1 group—good (normal) in 8 patients, poor in 1, and NLP in 1; non-NF-1 group—good in 5 patients, moderate in 2, poor in 4, and NLP in 3. At the end of follow-up, in the NF-1 group, vision was good in 8, moderate in 1 and NLP in 1; findings in the non-NF-1 group were good in 4, and moderate in 1, poor in 4, and NLP in 4. A deterioration in **v**isual acuity was noted in 1/10 patients with NF-1, from 20/320 to hand motion (HM), and 5/13 patients without NF-1. Initial average score on 10-plate Ishihara color vision test was 8.4 in the NF-1 group and 5.9 in the non-NF-1 group. At the end of follow-up, the respective averages were 9.3 and 5.6.

### Neuroimaging

On repeated neuroimaging, at the end of the follow-up period, the tumor remained stable in 8 patients in each group, progressed in 2 patients with NF-1 and 3 without NF-1, and regressed in 2 patients, both without NF-1, respectively. One patient without NF-1 and tumor progression had an intralesional hemorrhage.

### OCT Scanning

Mean age at the first OCT examination in the NF-1 and non-NF-1 groups was 14.9 years and 12.9 years, respectively. Mean RFNL thickness at the first examination was 85.4 μm in the NF-1 group (data available for 19 eyes) and 65 μm in the non-NF-1 group (data available for 23 eyes), *p* = 0.0025. At the last follow-up, RNFL measured 72.6 μm in the NF-1 group (data available for 8 eyes) and 60.1 μm in the non-NF-1 group (data available for 21 eyes), but this difference was not statistically significant (*p* = 0.06).

OCT was repeated in 15 patients, 4 with NF-1 and 11 without NF-1, at a median of 2 years from the first OCT evaluation. Thinning of the RNFL was noted in 3 patients with NF-1 (5 eyes) and 8 patients without (11 eyes). Further analysis of the patients with RNFL thinning revealed clinical deterioration in 2 of the patients with NF-1 and 6 of the patients without non-NF-1. Clinical deterioration occurred at various intervals during the course of follow-up. Tables 2, 3 show the first and last OCT results for each patient alongside the changes in visual function. One representative patient from each group for whom complete data on all evaluations were available are described below.

**Table 2:** RNFL thickness values in OCT by quadrant in children without NF-1 and the corresponding optic nerve function findings.

**Pt. no**.		**RNFL thickness (μm) 1**^****st****^ **OCT**					**RNFL thickness (μm) last OCT**					**OPG Location**	**Initial optic nerve function (Last)**				
		**Ave**	**SQ**	**IQ**	**NQ**	**TQ**	**Ave**	**SQ**	**IQ**	**NQ**	**TQ**		**VA (LogMar)**	Color vision^*^	**RAPD**	**Disc**	**VF**
1	RE	45	34	52	55	42	38	38	47	35	51	OT, CG	0.4 (0.6)	0.0 (0.0)	+ (+)	Pale (Pale)	HOMHEM (HOMHEM)
	LE	55	67	73	43	38	52	64	75	45	29		0.2 (0.1)	1.0 (0.9)	– (–)	Pale (Pale)	HOMHEM (HOMHEM)
2	RE	58	89	47	49	47	70	104	76	48	51	CG	0.0 (0.0)	YO (10)	– (–)	Normal (Pale)	Normal (Bitemporal)
	LE	–	–	–	–	–	51	53	56	46	48		0.0 (1.0)	YO (0)	– (+)	Normal (Pale)	Normal (Bitemporal)
3	RE	57	73	45	59	54	–	–	–	–	–	OT, HT	0.0 (0.0)	0.6 (0.5)	– (–)	Normal (Pale)	HOMHEM (HOMHEM)
	LE	68	101	90	39	45	–	–	–	–	–		0.1 (0.0)	0.6 (0.5)	– (+)	Atrophy (Atrophy)	HOMHEM (HOMHEM)
4	RE	70	–	–	–	–	69	–	–	–	–	ON, CG, HT	NLP (NLP)	YO (0.0)	+ (+)	Pale (Atrophy)	YO (None)
	LE	72	99	102	51	35	67	88	93	46	43		0.0 (0.0)	YO (1.0)	– (–)	Normal (Pale)	YO (Temporal)
5	RE	42	37	53	32	46	52	56	53	43	56	ON, CG	CF (CF)	0.0 (0.0)	+ (+)	Atrophy (Atrophy)	HOMHEM (HOMHEM)
	LE	64	69	105	31	54	64	76	86	41	54		0.0 (0.0)	1.0 (1.0)	– (–)	Pale (Pale)	HOMHEM (HOMHEM)
6	RE	48	49	48	56	37	48	44	51	51	47	CG, HT	0.1 (0.2)	1.0 (0.9)	– (+)	Pale (Atrophy)	Bitemporal (Bitemporal)
	LE	47	48	53	58	30	46	45	50	47	40		0.1 (0.0)	1.0 (0.9)	– (–)	Pale (Pale)	Bitemporal (Bitemporal)
7	RE	64	84	80	42	50	51	51	46	66	39	CG, HT	1.1 (1.2)	0.0 (0.0)	+ (+)	Pale (Atrophy)	Temporal (HOMHEM)
	LE	59	83	68	43	44	60	76	70	49	43		0.1 (0.1)	0.2 (1.0)	– (–)	Pale (Atrophy)	Normal (HOMHEM)
8	RE	52	48	78	45	37	46	43	67	44	32	HT	0.4 (1.0)	0.3 (0.0)	– (–)	Atrophy (Atrophy)	Nasal (Nasal)
	LE	58	95	60	44	31	47	80	50	32	30		1.3 (1.4)	0.0 (0.0)	+ (+)	Atrophy (Atrophy)	Nasal (Nasal)
9	RE	83	73	119	94	47	65	63	92	64	42	HT	0.0 (0.1)	1.0 (1.0)	– (–)	Normal (Pale)	Normal (HOMHEM)
	LE	75	68	125	56	52	59	59	80	56	39		0.0 (0.1)	1.0 (1.0)	– (–)	Normal (Pale)	Normal (HOMHEM)
10^**^	RE	–	–	–	–	–	–	–	–	–	–	CG	LP (NLP)	0.0 (0.0)	+ (+)	Atrophy (Atrophy)	None (None)
	LE	55	75	56	42	47	-	-	-	-	-		0.1 (0.1)	0.8 (0.8)	– (–)	Pale (Pale)	Normal (Normal)
11^**^	RE	117	139	144	99	89	101	122	131	88	69	ON	0.0 (0.0)	1.0 (1.0)	– (–)	Normal (Normal)	Normal (Normal)
	LE	–	–	–	–	–	–	–	–	–	–		NLP (NLP)	0.0 (0.0)	+ (+)	Pale (Pale)	None (None)
12	RE	96	103	100	133	48	85	99	72	50	42	OT, HT	0.0 (0.0)	1.0 (1.0)	– (–)	Pale (Pale)	HOMHEM (HOMHEM)
	LE	70	93	118	36	36	75	112	102	48	38		0.0 (0.0)	1.0 (1.0)	+ (+)	Pale (Pale)	HOMHEM (HOMHEM)
13	RE	83	109	112	52	60	67	76	94	48	49	CG, HT	0.0 (0.0)	1.0 (1.0)	– (–)	Normal (Pale)	Bitemporal (Temporal)
	LE	57	79	60	49	40	50	57	50	47	48		0.7 (NLP)	0.5 (0.0)	+ (+)	Pale (Atrophy)	Bitemporal (None)

*OCT, optical coherence tomography; Ave, average; SQ, superior quadrant; IQ, inferior quadrant; NQ, nasal quadrant; TQ, temporal quadrant; VA, visual acuity; YO, too young to perform; RAPD, relative afferent pupillary defects; VF, visual fields; HOMHEM, homonymous hemianopia; ON, Optic nerve glioma; OT, Optic tract glioma; CG, Chiasmatic glioma; HT, Hyopthalamic glioma*.

* *Ishihara color plates detected*.

** *In some NLP eyes, OCT scanning was of poor quality and not included*.

**Table 3:** RNFL thickness values in OCT by quadrant in children with NF-1 and the corresponding optic nerve function findings.

**Pt. no**.		**RNFL thickness (μm) 1st OCT**					**RNFL thickness (μm) last OCT**					**OPG Location**	**Initial optic nerve function (Last)**				
		**Ave**	**SQ**	**IQ**	**NQ**	**TQ**	**Ave**	**SQ**	**IQ**	**NQ**	**TQ**		**VA (LogMar)**	Color vision^*^	**RAPD**	**Disc**	**VF**
1	RE	45	61	56	35	31	41	49	53	34	27	ON, CG	NLP (NLP)	0.0 (0.0)	+ (+)	Atrophy	None
	LE	–	–	–	–	–	43	62	49	35	23		0.3 (0.3)	0.5 (0.3)	– (–)	Atrophy	Temporal (Temporal)
2	RE	97	107	130	85	68	78	71	119	62	63	CG, HT	0.0 (0.0)	0.8 (1.0)	– (–)	Normal (Normal)	Normal (Normal)
	LE	96	104	130	95	53	82	78	123	50	79		0.0 (0.0)	1.0 (1.0)	– (–)	Normal (Normal)	Normal (Normal)
3	RE	110	130	125	96	88	–	–	–	–	–	ON	0.0 (0.1)	1.0 (1.0)	– (–)	Normal (Normal)	Normal (Normal)
	LE	102	122	105	96	84	–	–	–	–	–		0.0 (0.1)	1.0 (1.0)	– (–)	Normal (Normal)	Normal (Normal)
4	RE	86	98	120	67	61	90	113	118	66	63	ON, CG	0.0 (0.0)	1.0 (1.0)	– (–)	Normal (Normal)	Normal (Normal)
	LE	71	81	95	67	41	69	74	99	61	40		0.0 (0.0)	1.0 (1.0)	– (–)	Normal (Normal)	Normal (Nasal)
5	RE	76	108	91	56	52	–	–	–	–	–	ON	0.0 (0.1)	YO (1.0)	– (–)	Normal (Pale)	Normal (Normal)
	LE	73	81	104	56	54	–	–	–	–	–		0.0 (0.0)	YO (1.0)	– (–)	Normal (Pale)	Normal (Normal)
6	RE	60	73	73	52	42	49	51	59	52	34	ON, OT, CG, HT	0.2 (0.2)	1.0 (0.5)	– (–)	Pale (Atrophy)	Bitemporal (Temporal)
	LE	57	70	53	56	49	48	51	50	50	40		1.2 (HM)	0.3 (0.0)	– (+)	Atrophy (Atrophy)	Bitemporal (None)
7	RE	86	116	100	71	58	78	107	86	48	72	OT	0.2 (0.0)	1.0 (1.0)	– (–)	Normal (Normal)	Normal (Bitemporal)
	LE	89	111	96	78	72	87	110	97	70	72		0.0 (0.0)	1.0 (1.0)	– (–)	Normal (Normal)	Normal (Bitemporal)
8	RE	101	109	141	85	68	–	–	–	–	–	ON, OT, CG, HT	0.0 (0.0)	1.0 (1.0)	– (–)	Normal (Normal)	Normal (Normal)
	LE	99	130	132	69	64	–	–	–	–	–		0.0 (0.0)	1.0 (1.0)	– (–)	Normal (Normal)	Normal (Normal)
9	RE	113	173	111	67	102	–	–	–	–	–	ON, CG	0.0 (0.0)	1.0 (1.0)	– (–)	Normal (Normal)	Normal (Normal)
	LE	47	51	59	42	35	–	–	–	–	–		0.0 (0.0)	1.0 (1.0)	+ (+)	Atrophy (Atrophy)	Nasal (Nasal)
10	RE	106	110	137	88	64	–	–	–	–	–	ON, OT, CG, HT	0.0 (0.0)	0.8 (1.0)	– (–)	Normal (Normal)	Normal (Normal)
	LE	108	133	137	95	67	–	–	–	–	–		0.0 (0.0)	0.8 (1.0)	– (–)	Normal (Normal)	Normal (Normal)

*OCT, optical coherence tomography; Ave, average; SQ, superior quadrant; IQ, inferior quadrant; NQ, nasal quadrant; TQ, temporal quadrant; VA, visual acuity; YO, too young to perform; RAPD, relative afferent pupillary defects; VF, visual fields; HOMHEM, homonymous hemianopia; ON, Optic nerve glioma; OT, Optic tract glioma; CG, Chiasmatic glioma; HT, Hyopthalamic glioma*.

* *Ishihara color plates detected*.

### Representative Cases

**Figure 1:**
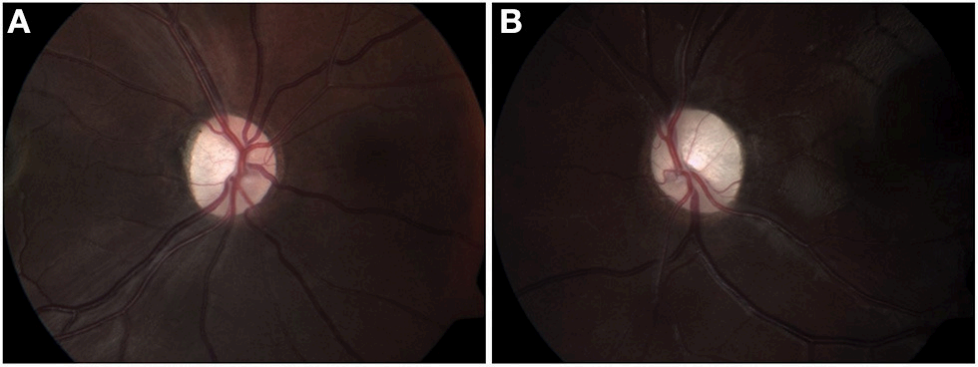
Fundus color images (2012) of right **(A)** and left **(B)** optic discs, showing mild pallor on the right **(A)**, and disc atrophy with advanced cupping on the left **(B)**.

**Figure 2:**
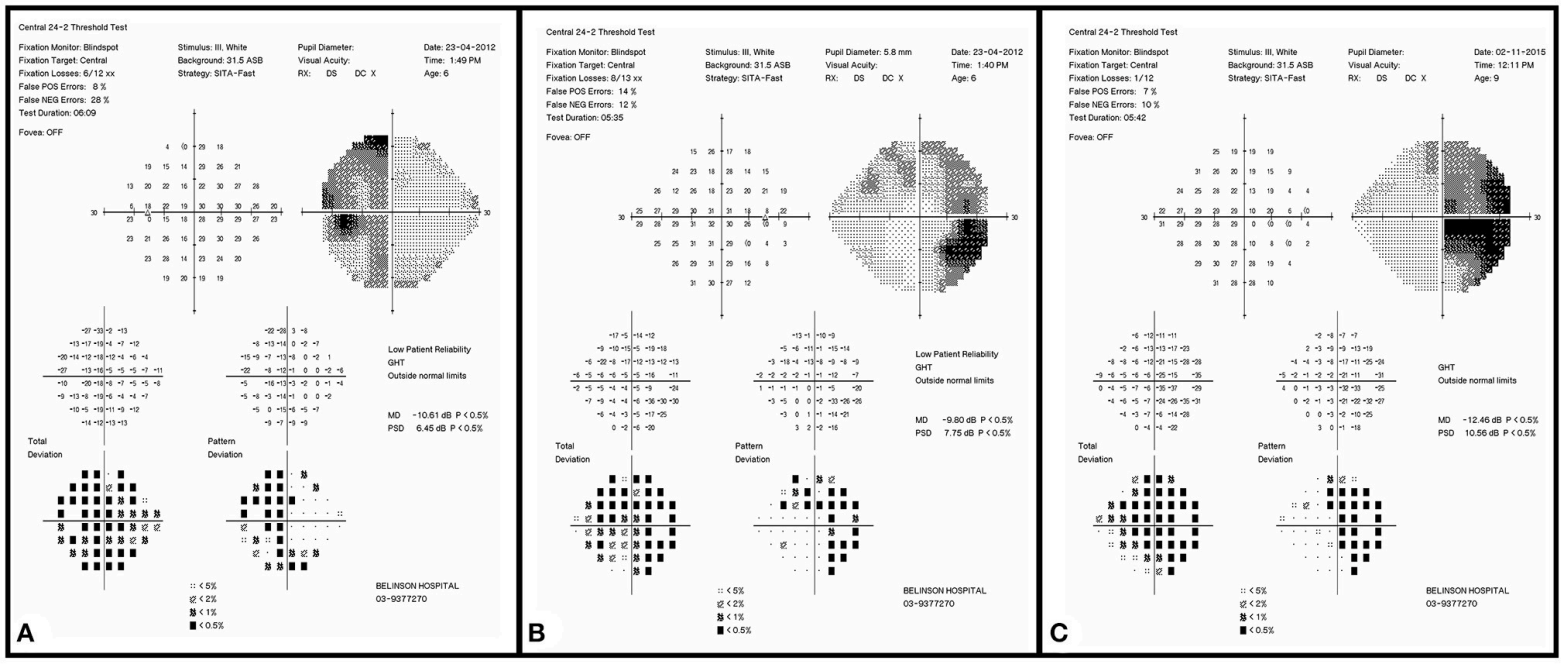
Initial visual field examination (2012) demonstrating bitemporal hemianopia. Left eye **(A)** and right eye **(B)**. Final visual field examination (2015) demonstrating worsening in the right eye **(C)**. The left final visual acuity was too poor for visual field testing.

**Figure 3:**
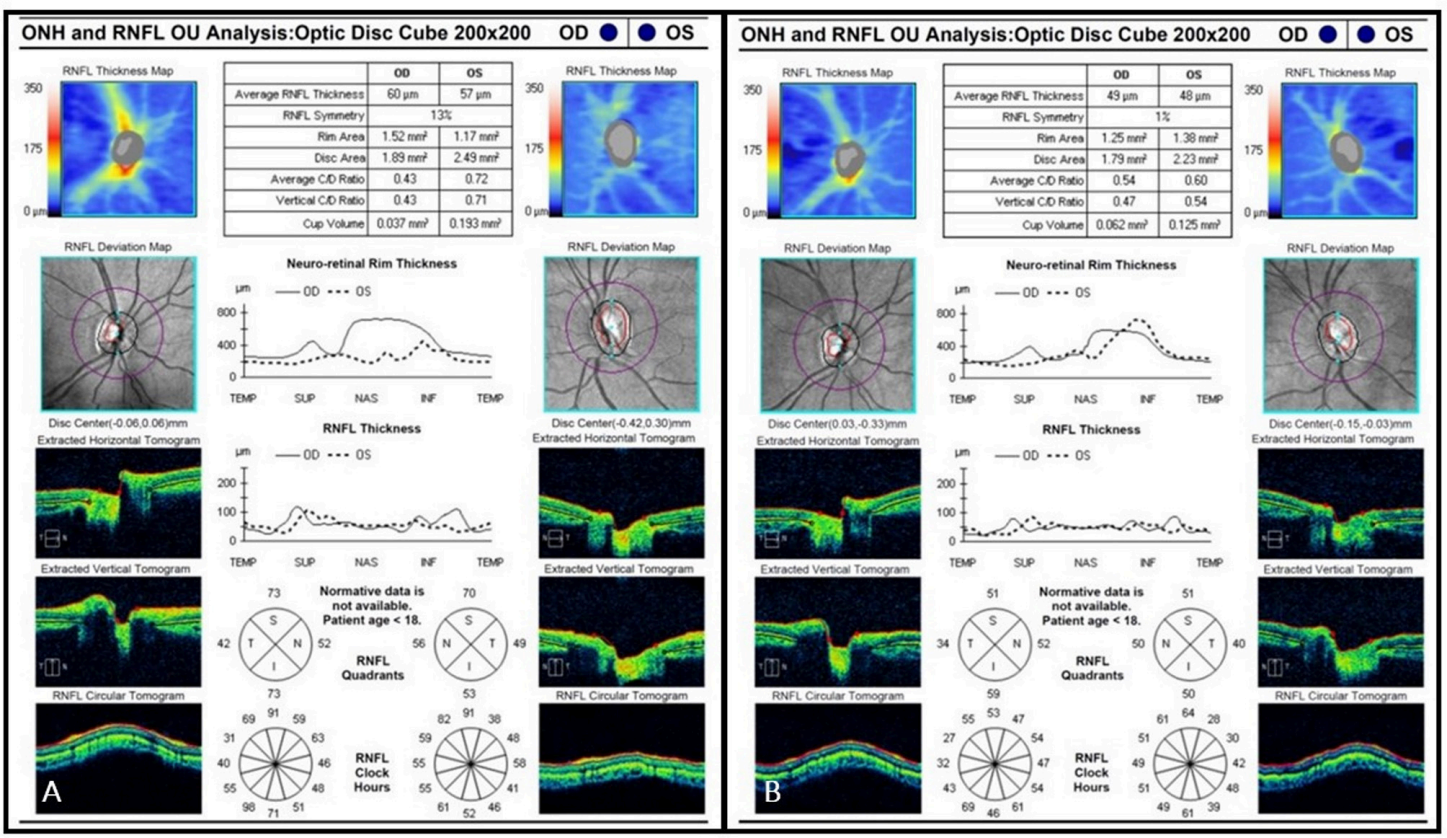
**(A)** Initial OCT scan (2012) demonstrating bilateral RNFL thinning. Average RNFL thickness is 60 μm on the right and 57 μm on the left. **(B)** Final OCT scan (2015) demonstrating progressive bilateral RNFL thinning. Average RNFL thickness is 49 μm on the right and 48 μm on the left.

**Figure 4:**
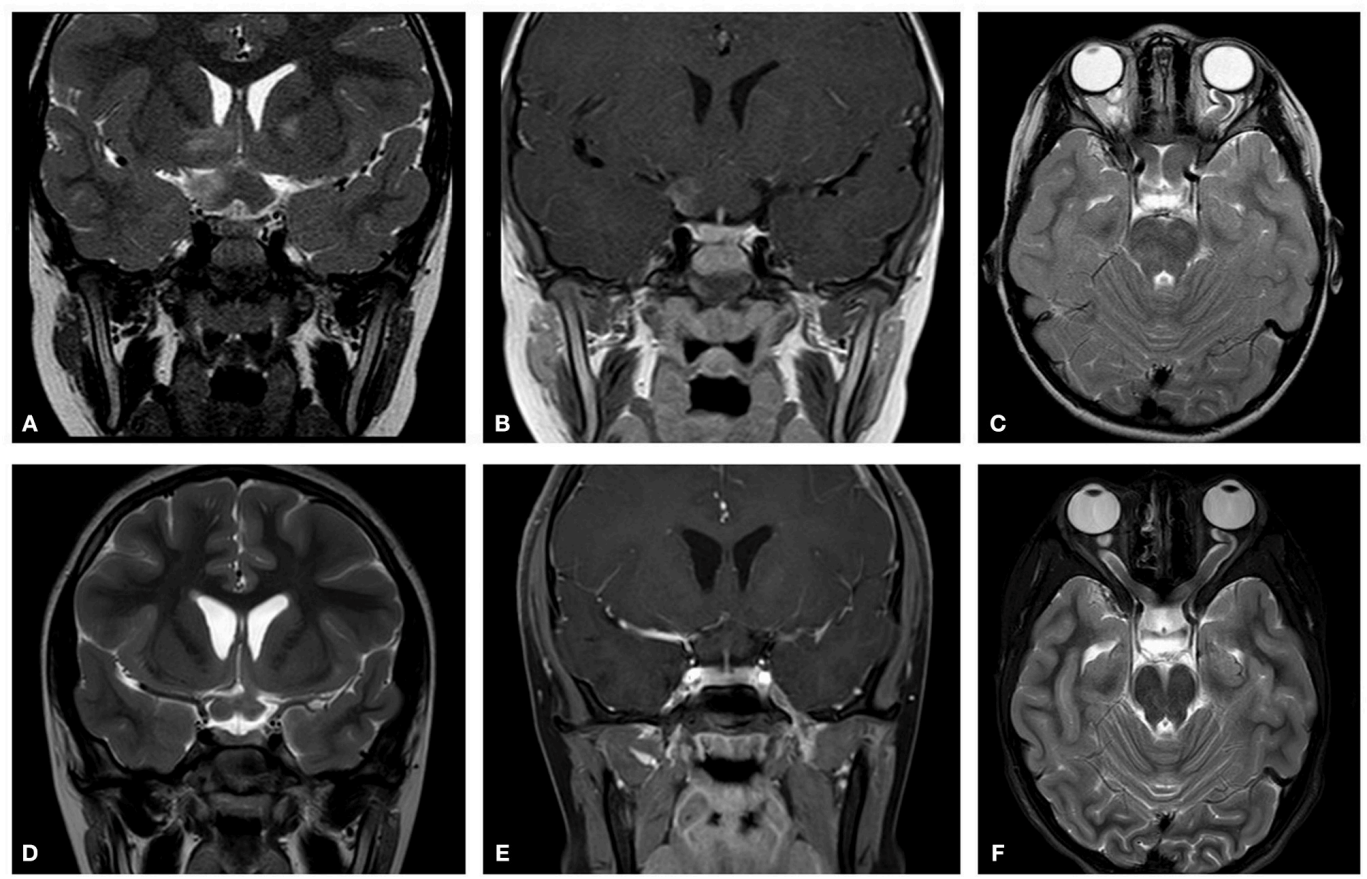
Initial MRI (2012). Showing chiasmatic glioma with bilateral optic nerve involvement. **(A)** A T2 coronal section shows a thickening of the chiasm with high signal within the right chiasm and high signal within the right hypothalamus extending into the basal ganglia. **(B)** A T1 coronal section post contrast shows right chiasmatic enhancement. **(C)** In a T2 axial section a bilateral kinking of the optic nerve is evident with pre-chiasmatic thickening, and a left optic nerve thickening. Post treatment (2015) **(D)** A T2 coronal section demonstrates that the chiasm is less thickened and that there is no high signal with the right hypothalamus. **(E)** A T1 coronal section post contrast shows no enhancement of the thickened chiasm. **(F)** A T2 axial section reveals bilateral kinking of the optic nerve, but with reduced thickening of the left optic nerve as compared to the initial scan.

A 6-year-old girl with café au lait lesions was found to have left optic disc pallor on routine screening. She was referred for neuro-ophthalmologic evaluation for suspected NF-1. Visual acuity was 20/32 in the right eye and 20/320 in the left, and Ishihara color vision score was normal on the right and low (3/10 plates) on the left, without RAPD. Lisch nodules were observed on the right iris. Fundus examination revealed right mild optic disc pallor and left optic disc atrophy with advanced cupping (Figures 1A,B). Visual field examination revealed bitemporal hemianopia (Figures 2A,B), and OCT revealed bilateral RNFL thinning (Figure 3A). MRI showed a chiasmatic glioma with bilateral optic nerve involvement (Figures 4A–C). The patient was treated with chemotherapy sessions. On follow-up, both optic discs were further atrophied, and left RAPD developed with a decrease in left visual acuity to HM. Ishihara color vision score decreased, as well as the visual fields (Figure 2C). No progression was noted on MRI (Figures 4D–F) relative to the OCT findings of progression of RNFL thinning (Figure 3B).

**Figure 5:**
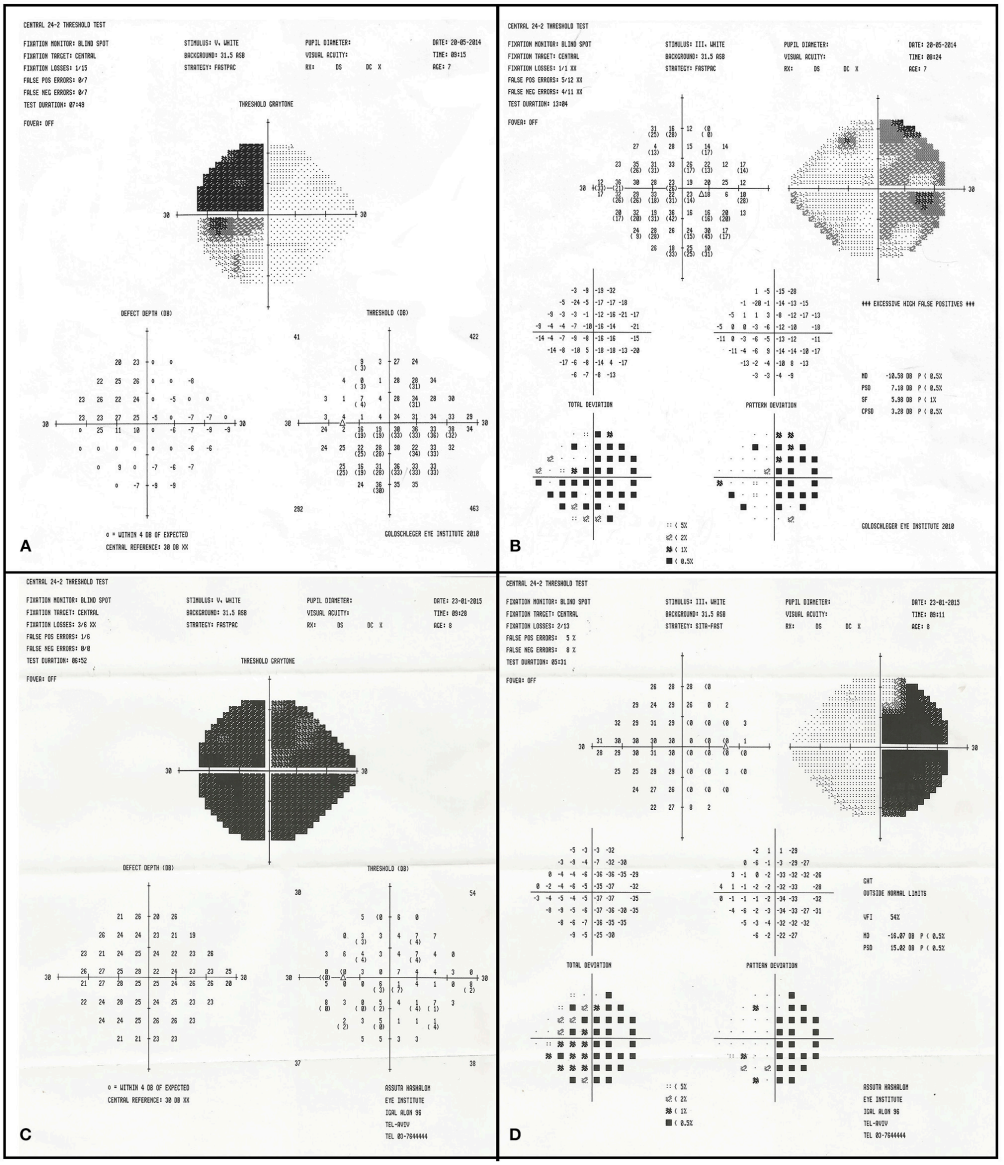
Initial visual field examination (2014) demonstrating bitemporal hemianopia. There is a deeper scotoma of the left upper temporal quadrant **(A)** than on the right **(B)**. Final visual field examination (2015) demonstrating diminished residual upper nasal field on the left **(C)** and worsening in the right eye **(D)**.

**Figure 6:**
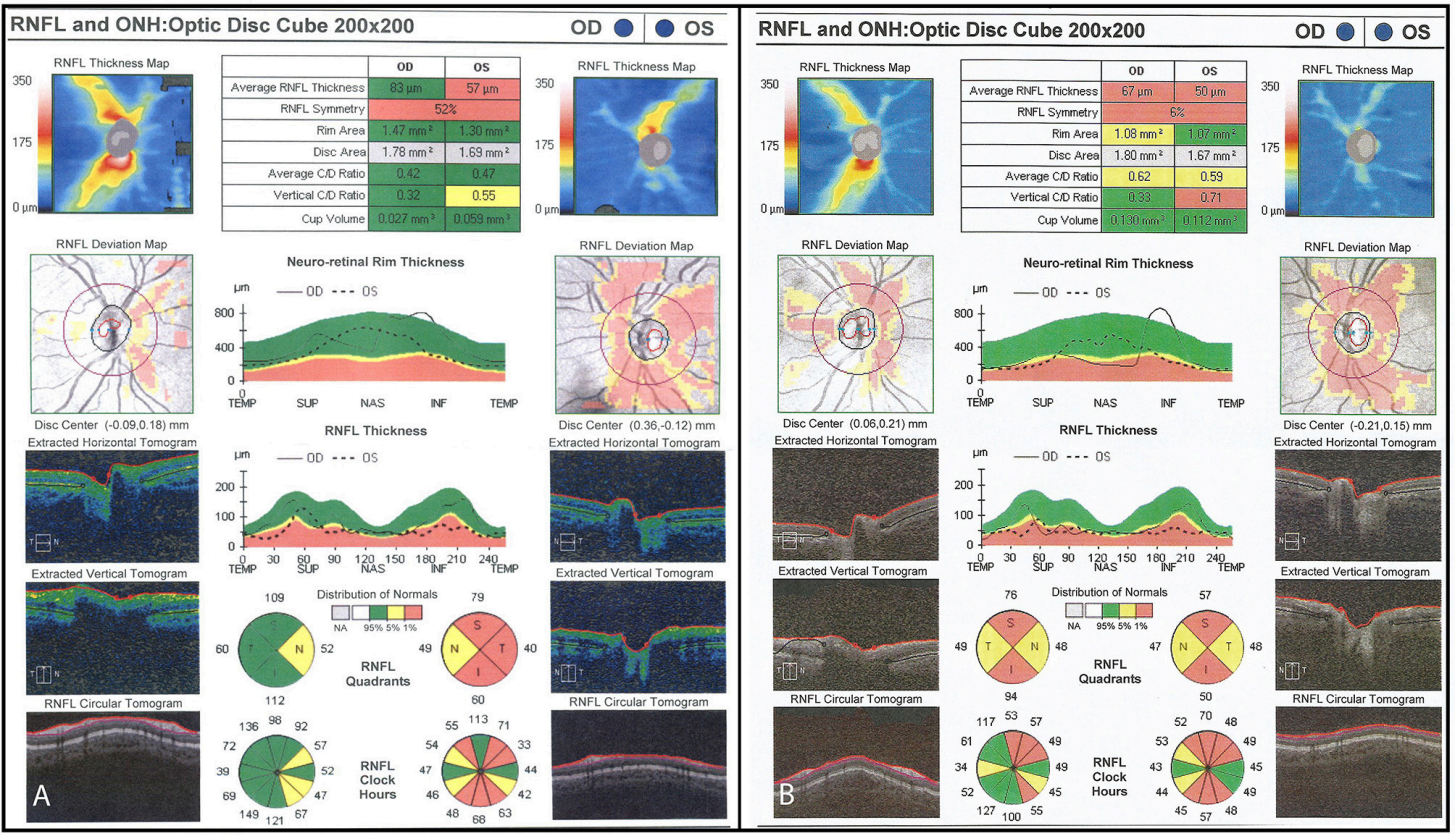
**(A)** Initial OCT scan (2014) demonstrating reduced RNFL thickness on the left optic nerve as compared to the right. Average RNFL thickness is 83 μm on the right and 57 μm on the left. **(B)** Final OCT scan (2015) demonstrating progressive bilateral RNFL thinning. Average RNFL thickness is 67 μm on the right and 50 μm on the left.

**Figure 7:**
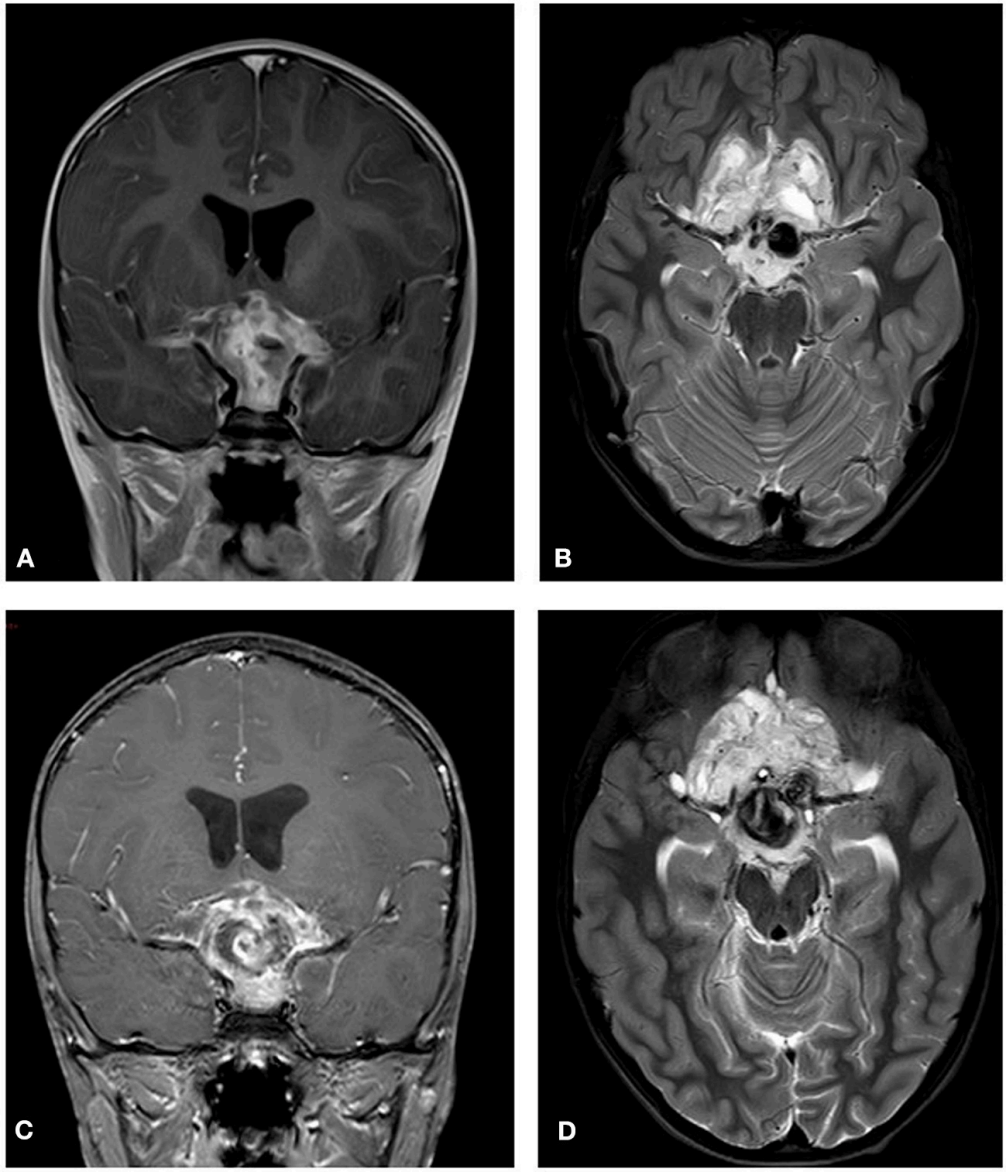
Initial MRI (2014). A hypothalamic mass is demonstrated. **(A)** A T2 post contrast coronal scan shows a high signal enhancing lesion within the sellar and suprasellar region encasing the chiasm. **(B)** A T2 axial section shows a high signal large heterogenous chiasmatic and suprasellar lesion, with a left supra-clinoid carotid aneurysm. Post treatment (2015). **(C)** A T2 coronal section shows a smaller lesion, with a large hemorrhagic intralesional focus which might be secondary to partial rupture of the aneurysm within the lesion. **(D)** A T2 axial section without contrast.

A 7-year-old boy was referred for evaluation because of abnormal findings on a school visual acuity screening test. Visual acuity was 20/20 in the right eye and 20/100 in the left. Ishihara color test score was normal on the right and low (5 of 10) on the left, and visual field examination revealed bitemporal hemianopia with a deeper scotoma on the left upper temporal quadrant (Figures 5A,B). Left RAPD and left optic pallor were noted. OCT revealed reduced RNFL thickness on the left compared to the right (Figure 6A). Initial MRI revealed a hypothalamic mass (Figures 7A,B). The diagnosis was non-NF-1 optic glioma. Eight months after treatment was started, there was intra-tumoral bleeding (Figures 7C,D). Emergency surgery was performed, and a shunt was inserted. Vision in the left eye decreased to HM. Follow-up visual field (Figures 5B,C) and OCT (Figure 6B) examinations showed disease progression in the right eye with maintenance of central visual acuity. The patient is currently undergoing rehabilitation after a long hospitalization. He has nasal vision (best corrected visual acuity 20/20) in the right eye and NLP in the left. Tumor regrowth was noted on repeated MRIs since the hemorrhage.

### Association of OCT and Visual Function Results

On statistical analysis, thinning of the RNFL on OCT in both the initial and last scan was found to be significantly associated with worse visual acuity (higher logMAR score). This was true both overall (*P* < 0.001) and by quadrant (*P* ≤ 0.05) in both groups (Tables 2, 3). A deterioration in OCT findings was also significantly associated with a decrease in color vision (Ishihara test) and worsening visual field findings (*P* < 0.05 for both). The presence of optic disc atrophy was significantly associated with findings on optic disc measurements by OCT (*P* < 0.05).

## Discussion

OPG compression in the optic nerve, chiasm, or optic tract poses a risk of Wallerian degeneration and subsequent thinning of the RNFL. Owing to the difficulty in assessing visual function in young children, OCT has been suggested as a surrogate tool for optic nerve evaluation (33–35). In the present study, OCT was applied to measure RNFL thickness in children with OPG, with and without NF-1, and the results were compared to visual function tests and neuroimaging. We found that the OCT measurements of the RNFL thickness corresponded with clinical parameters of visual function, especially visual field defects and optic atrophy. The clinical results did not correlate with neuroimaging findings, which showed a mainly stable state of the glioma throughout follow-up. Our neuroimaging findings agree with reports in the literature that OPGs rarely regress regardless of treatment (2, 4, 17).

This study also highlights differences in the course of OPG between children with and without NF-1. Clinical deterioration occurred in some children, mainly in the non-NF-1 group. These findings agree with earlier reports showing that in children with NF-1, OPGs are generally asymptomatic and clinical deterioration is relatively rare (14). The WHO recommends that visual assessment be performed in children with NF-1 every year to age 17 years and MRI in patients presenting with signs of OPG (13). Previous studies have shown monocular nystagmus to be a common presenting sign in infants (36). In our study, the patients without NF-1 were tested for OPG when symptoms or signs started, most commonly visual disturbances. It is generally accepted that after the onset of OPG symptoms, patients without NF-1 need to be reevaluated by MRI every 3 months in the first year and at least once annually thereafter (13, 14). Treatment in this group was often based on tumor-induced symptoms and signs, and findings on follow-up imaging were stable in most cases. Nevertheless, clinical deterioration in the optic nerve function was evident in 10 patients (76.9%), despite 8 patients (61.5%) having stable MRI findings. In 6, there was matching progressive thinning on OCT; in 3 patients, either initial or follow-up OCT data were unavailable for the eye with progression; and in 1 patient, RNFL thinning was already noted on the baseline OCT scan. Follow-up imaging, especially MRI, is important in children with OPG to measure tumor size, but as shown in this study, changes in tumor size (either reduction or enlargement) are not always correlated with the clinical signs. Therefore, clinical examination with OCT measurements can be valuable in determining the treatment strategy.

Because OPGs often affect young patients, clinical follow-up can be complicated by their young age and poor verbal communication. We found stationary OCT to be applicable even in young children, though it was more accessible for children older than 5 years. Hand- held OCT, as described in the literature, if available, might be used even in younger age groups (26, 29). In our cohort, OCT usually demonstrated a normal optic nerve with average RNFL thickness in association with normal visual function. There was also a good association in cases of abnormal RNFL thickness with impaired visual functions, mainly bitemporal or homonymous field defects, reduced visual acuity, or reduced color vision. OCT also made the follow-up evaluation easier and more accurate than computerized analysis of the visual fields which demands good cooperation from the child and is often unfeasible before age 6 years. In an earlier large study of the use of OCT in young children with gliomas, the authors found that measurements of RNFL thickness acquired under sedation could differentiate those with and without vision loss (26). In later studies, the same authors measured RNFL in the macula and found that reduced thickness was significantly associated with lower visual acuity/ higher logMAR scores (*P* < 0.001) (28, 31, 37). However, we cannot directly compare these findings with our study in which RNFL was measured in optic disc fibers. Furthermore, sedation carries risks and increases the risk to young children who also need sedation for the MRI. Hand-held OCT performed during MRI sedation can resolve this issue.

We found that RNFL thickness was reduced mostly in the temporal quadrant, followed in order by the nasal, superior, and inferior quadrant, as indicative of RNFL loss in pathological nerves. This is in agreement with Chan and Miller (38), who found the temporal sector to be thinnest in severe optic neuropathies. When the glioma was in a more posterior location, such as the optic chiasm, atrophy was greatest in several nerves in the nasal area. This pattern of damage may result from a particular vulnerability of macular axons to compression in this site as a consequence of the location of the fibers within the optic nerve.

There are prior reports on the differences between sporadic (non-NF-1) gliomas and NF-1 associated gliomas, but only a few evaluated the OCT measurements in these two groups. Avery et al. reported longitudinal changes in OCT and correlation to visual loss in children with OPGs, demonstrating a correlation between RNFL thinning and visual loss (30). Similar to our study, they found that sporadic gliomas were more likely to cause vision loss than NF-1 associated gliomas, but they did not directly compare the NF-1 and non-NF-1 patients on OCT.

This study is limited by the retrospective design and the small number of patients for whom OCT measurements were available, specifically on follow-up of the NF-1 group. In addition, the macular ganglion cell layer analysis was not routinely acquired and therefore could not be evaluated.

This study was performed in a tertiary medical center staffed by experienced pediatric neuro-ophthalmologists and equipped with modern devices. OCT may be particularly useful for assessing optic nerve function in more peripheral medical centers that lack these means.

Further studies are still needed to determine the relationship between the OCT results and the anatomic location of OPGs and the influence of glioma involvement on the optic nerve thickness. We believe larger, longitudinal studies with more modern and accurate RNFL and macular ganglion cell layer analysis would be useful to determine the value of OCT as a surrogate marker of the clinical progression of OPG. This could reduce the frequency of MRI studies. Furthermore, OCT studies may overcome the poor relationship between changes in tumor size on MRI and visual outcomes (30).

## Ethics Statement

This study was carried out in accordance with the recommendations of the Institutional Review Board Ethics Committee at Rabin Medical Center, Israel. Written informed consent was waived by the ethics committee as this is a retrospective study with de-identified patient information. The protocol was approved by the Institutional Review Board Ethics Committee at Rabin Medical Center, Israel.

## Author Contributions

NG-C, RC, and JL were responsible for conception and design. AZ, SS, RC, HT, and SM were responsible for acquisition of data. AZ, SS, RC, and HT, SM, and NG-C were responsible for analysis and interpretation of data. AZ, SS, HT, JL, and NG-C drafted the article. RC and SM revised the article for intellectual content. All authors approved the final version.

### Conflict of Interest Statement

The authors declare that the research was conducted in the absence of any commercial or financial relationships that could be construed as a potential conflict of interest.
